# Pangenome and pan-transcriptome analyses reveal evolutionary diversification of the *GPAT* gene family in soybean

**DOI:** 10.3389/fpls.2026.1988876

**Published:** 2026-10-14

**Authors:** Quanlong Liu, Jiaqi Lian, Yao Liu, Yingmei Peng, Juan He

**Affiliations:** 1 School of Life Science and Technology, Northwestern Polytechnical University, Xi’an, Shaanxi, China; 2 School of Life Sciences, North China University of Science and Technology, Tangshan, China

**Keywords:** soybean, *GPAT* gene family, pangenome, pan-transcriptome, gene duplication

## Abstract

Glycerol-3-phosphate acyltransferases (*GPAT*s) participate in plant lipid metabolism, yet their intraspecific evolutionary and transcriptional diversity in soybean remains incompletely characterized. Here, we integrated a survey of 1,011 plant species with pangenome and pan-transcriptome analyses of 29 soybean accessions. We identified 15,799 *GPAT* genes across plants and 1,329 across soybean accessions; the latter were classified into three subfamilies, with 42–47 genes per accession. Among soybean *GPAT* genes, 1,317 were assigned to 47 orthogroups, comprising 19 core and 28 dispensable groups, of which 22 were softcore. Whole-genome/segmental duplication accounted for 86.38% of soybean *GPAT* genes, and 94.04% of homologous gene-pair comparisons exhibited Ka/Ks < 1, indicating predominant purifying selection. Whole-genome/segmental duplicates were more prevalent among core genes, which also showed lower median Ka/Ks ratios than dispensable genes. Subfamily III contained the highest proportion of dispensable genes, whereas subfamily II exhibited the highest mean Ka/Ks ratio, revealing distinct patterns of gene-content and coding-sequence divergence. Expression profiling across eight tissues revealed transcriptional divergence within subfamilies and greater expression stability of core pangenes across accessions. Co-expression networks shared 27 nodes and 25 edges among cultivars, landraces, and wild soybeans, with both core and dispensable members occupying highly connected positions. Together, these results highlight the coexistence of evolutionary conservation, gene-content variation, and transcriptional divergence within the soybean *GPAT* family. These findings provide candidate GPAT genes and testable hypotheses for future functional studies related to soybean lipid traits and stress tolerance.

## Introduction

Soybean (*Glycine max L.*) is one of the most important food, oilseed, and economic crops worldwide. It plays a major role in global food security and agricultural production (Singer et al., 2023; Li et al., 2026). Soybean seeds are rich in isoflavones, unsaturated fatty acids, vitamins, minerals, and other bioactive compounds, which contribute to their high nutritional and health value (Singer et al., 2023). As a major global oilseed crop, soybean is also an important source of vegetable oil, while the co-produced soybean meal provides a valuable protein-rich feed resource, together supporting the economic importance of soybean production (Song et al., 2023). Refined soybean oil is widely consumed as an edible oil, whereas chemically modified soybean oil is increasingly used in non-food applications, including paints, coatings, cosmetics, biodiesel, lubricants, adhesives, and other bio-based materials (Clemente and Cahoon, 2009; Singh et al., 2023). With growing demand for high-quality edible oils and renewable bio-based industrial feedstocks, improving soybean oil yield, composition, and functional properties has become increasingly important. Therefore, understanding the mechanisms underlying lipid biosynthesis, accumulation, and genetic regulation is essential for both soybean nutritional improvement and the development of value-added industrial applications (Tong et al., 2025a; Li et al., 2026).

Glycerol-3-phosphate acyltransferases (*GPATs*) are key acyltransferases in plant glycerolipid metabolism. They catalyze the transfer of acyl groups to glycerol-3-phosphate (G3P), thereby generating intermediates required for downstream lipid biosynthesis (Jayawardhane et al., 2018; Waschburger et al., 2018). GPAT proteins generally contain several conserved acyltransferase motifs. The H(X)4D sequence in Motif I forms a major catalytic region, whereas Motifs II and III are mainly involved in substrate recognition and G3P binding. Motif IV is associated with acyl donor binding (Lewin et al., 1999; Turnbull et al., 2001). Land plant-specific *GPATs* have also undergone substantial functional diversification (Yang et al., 2012; Waschburger et al., 2018). In *Arabidopsis thaliana*, *GPAT4*, *GPAT6*, and *GPAT8* possess both sn-2 acyltransferase and phosphatase activities and mainly participate in cutin biosynthesis (Yang et al., 2010, 2012). By contrast, *GPAT5* and *GPAT7* are closely associated with suberin formation (Beisson et al., 2007; Yang et al., 2012). Differences in substrate preference and enzymatic activity further contribute to the functional specialization of *GPAT* members in distinct lipid metabolic pathways (Yang et al., 2012). In addition to lipid biosynthesis, *GPATs* are involved in plant growth, development, and stress responses (Jayawardhane et al., 2018). For example, GPAT-related pathways in *A. thaliana* are regulated by ABA and MYB transcription factors (Gou et al., 2017; Gully et al., 2024). *MYB107* promotes the expression of suberin biosynthetic genes, including *GPAT5*, while *GPAT5* and *GPAT7* participate in ABA-regulated root suberization (Gou et al., 2017; Gully et al., 2024). In soybean, *GmGPAT9–2* has been shown to possess *GPAT* activity and to affect fatty acid composition (Liu et al., 2022). More recently, the plastid-localized *GmGPAT1* was found to be strongly induced by salt stress, and its overexpression enhanced salt tolerance in soybean roots (Li et al., 2025b). Together, these findings indicate that *GPAT*s not only contribute to plant lipid metabolism but also play diverse roles in development and environmental adaptation.

With the rapid expansion of plant genomic resources, the *GPAT* gene family has been systematically identified and investigated in several model plants and economically important crops, including *A. thaliana*, rice, maize, cotton, and rapeseed (Waschburger et al., 2018; Cui et al., 2019; Zhu et al., 2019; Wang et al., 2020). These studies have revealed substantial variation in gene number, phylogenetic relationships, duplication patterns, and expression profiles. They have also improved our understanding of the roles of *GPAT* genes in lipid metabolism, plant growth and development, and environmental adaptation. However, studies of the *GPAT* family, including those in soybean, have largely relied on a single reference genome. This limits the detection of presence and absence variation, copy number variation, and potential functional divergence among different germplasm resources. A pangenome framework enables the separation of widely conserved core genes from dispensable genes and provides a more complete view of gene-content variation, duplication, and evolutionary diversification across soybean germplasm. In recent years, the development of soybean pangenome resources has greatly expanded our understanding of genomic diversity within this species (Liu et al., 2020; Torkamaneh et al., 2021; Li et al., 2025a). Studies based on multiple wild and cultivated soybean genomes have uncovered abundant nonreference sequences and variable genes that cannot be captured by a single reference genome. These resources provide an important basis for investigating gene family composition and evolution at the intraspecific level. Therefore, in this study, we systematically analyzed the soybean *GPAT* gene family from a pangenome perspective. We integrated phylogenetic analysis, presence and absence variation, copy number variation, gene duplication classification, selection pressure analysis, and Pan-transcriptome profiling to characterize the conservation, genetic variation, and evolutionary divergence of *GPAT* genes across soybean accessions. Our results provide a more comprehensive view of the intraspecific diversity and potential functions of the soybean GPAT family and generate candidate genes and hypotheses for future functional investigation.

## Results

### Large scale identification and analysis of the *GPAT* gene family

To investigate the distribution and expansion of the *GPAT* gene family across a broad evolutionary scale, we examined 1,011 plant species spanning algae to angiosperms and identified their *GPAT* family members. A total of 15,799 *GPAT* genes were identified (**Supplementary Table S1**). *GPAT* gene numbers varied substantially among species, with an average of 15.63 genes per species and a median of 13. Among the 752 dicot species, 12,315 *GPAT* genes were identified, with an average of 16.38 members per species and a range of 1 to 66 (**Figure 1**; **Supplementary Table S1**). A total of 2,625 *GPAT* genes were identified in 169 monocot species, with an average of 15.53 members per species and a range of 1 to 81. In contrast, algae generally contained fewer *GPAT* genes. The 44 algal species contained an average of only 6.75 *GPAT* genes, with gene numbers ranging from 1 to 10 (**Figure 1**; **Supplementary Table S1**). Bryophytes and gymnosperms contained an average of 10.83 and 10.11 *GPAT* genes per species, respectively. Magnoliids and early diverging angiosperms contained averages of 12.40 and 14.33 *GPAT* genes per species, respectively. Ferns contained an average of 17.25 *GPAT* genes per species (**Figure 1**; **Supplementary Table S1**). A Kruskal Wallis test revealed highly significant differences in *GPAT* gene number among plant groups, with H = 133.15 and P = 1.38 × 10^-25^ (**Figure 1**; **Supplementary Table S1**). Among all species examined, *Saccharum* spp. *R570* contained the largest number of *GPAT* genes, with 81 members, followed by *Onobrychis viciifolia* with 66 members and *Phyllanthus emblica* and *Hopea hainanensis* with 63 members each (**Figure 1**; **Supplementary Table S1**). Overall, *GPAT* genes are widely distributed across the plant kingdom, but their copy numbers vary considerably among evolutionary lineages and individual species. Angiosperms, particularly dicots and monocots, generally contain more *GPAT* members, suggesting that the *GPAT* family may have undergone varying degrees of gene expansion and functional diversification during the evolution of land plants, especially angiosperms.

**Figure 1:**
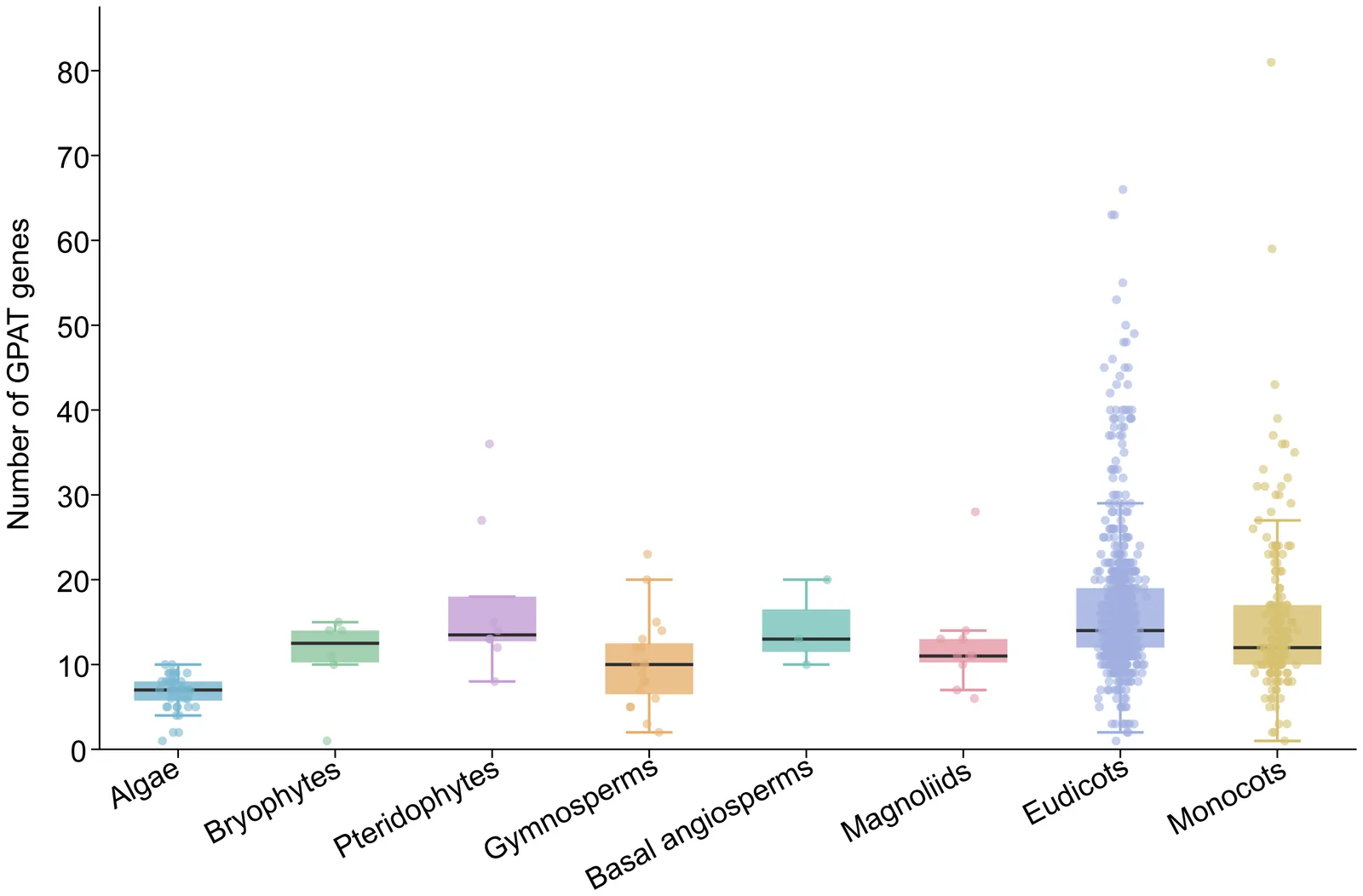
Distribution of *GPAT* gene family size across different plant groups. Each point represents the number of *GPAT* genes identified in an individual species, and different plant groups are indicated by different colors.

### Identification and phylogenetic analysis of the soybean *GPAT* gene family

To further characterize variation in *GPAT* gene content among soybean germplasm groups, we classified 29 soybean accessions into cultivars, landraces, and wild soybeans. A total of 1,329 *GPAT* genes were identified across these accessions (**Figure 2A**; **Supplementary Table S2**). The number of *GPAT* genes ranged from 42 to 47 per accession, with an average of 45.83. SoyC01 contained the fewest *GPAT* genes, with 42 members. In contrast, SoyC04, SoyC05, SoyC09, SoyC10, SoyC11, SoyC14, SoyL07, SoyL08, SoyL09, SoyW01, SoyW02, and W05 each contained 47 *GPAT* genes, representing the highest number among the analyzed accessions (**Figure 2A**; **Supplementary Table S2**). Overall, 25 of the 29 accessions contained at least 45 *GPAT* genes. Cultivars, landraces, and wild soybeans contained an average of 45.75, 45.56, and 46.75 *GPAT* genes per accession, respectively (**Figure 2A**; **Supplementary Table S2**). The variation in *GPAT* gene number was low within all three germplasm groups. The coefficient of variation was 3.24% in cultivars, 3.49% in landraces, and 1.07% in wild soybeans. Wild soybeans showed the lowest variation in *GPAT* gene number, whereas landraces showed slightly greater variation. Overall, the low coefficients of variation indicate that *GPAT* gene copy number is relatively stable across different soybean germplasm groups.

**Figure 2:**
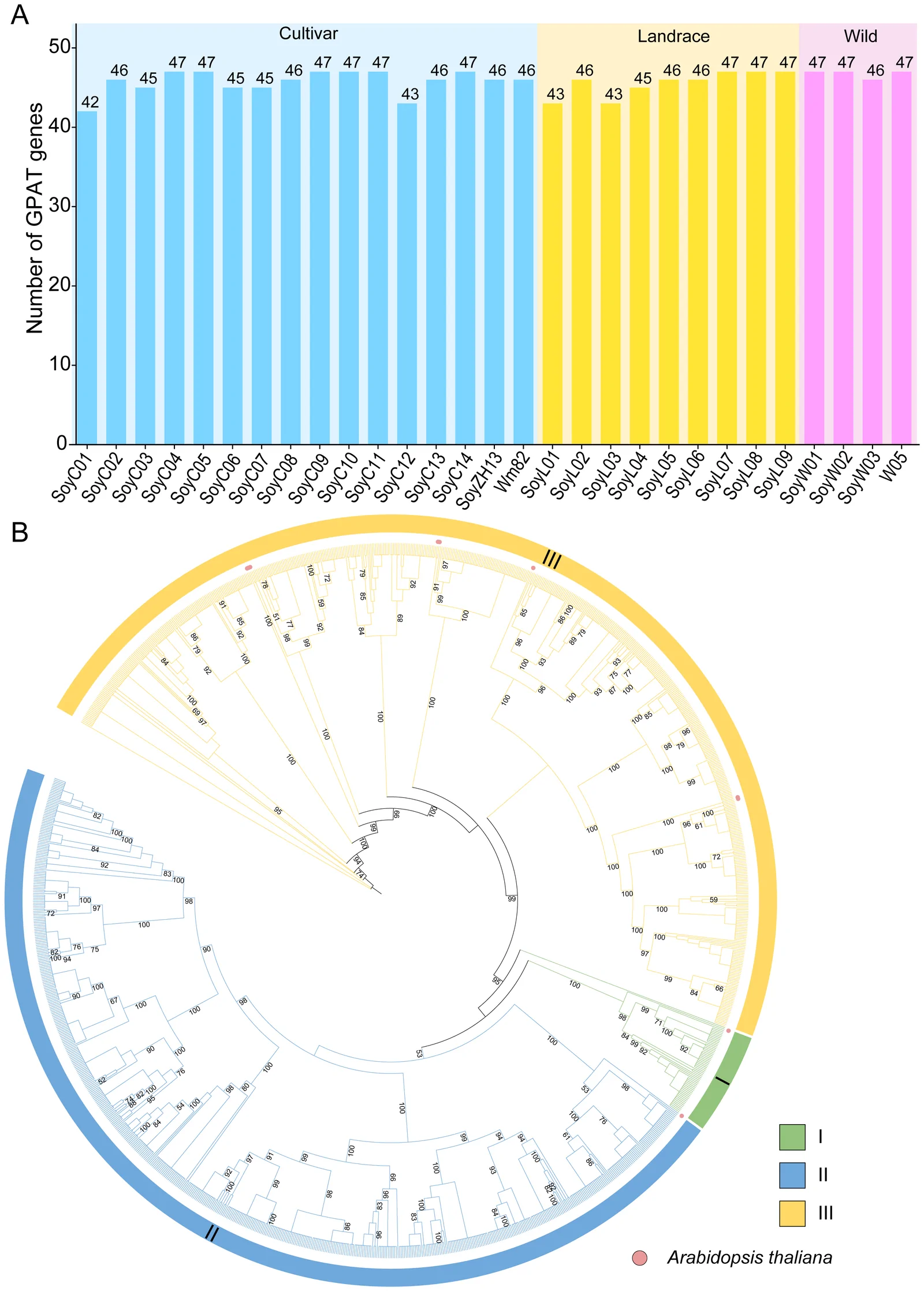
Distribution and phylogenetic relationships of *GPAT* genes across soybean accessions. **(A)** Number of *GPAT* genes identified in 29 soybean accessions, including cultivars, landraces, and wild soybeans. Different background colors represent the three soybean germplasm groups. **(B)** Phylogenetic relationships of soybean GPAT proteins and reference GPAT proteins from *A. thaliana*. The *GPAT* family was divided into three subfamilies, designated I, II, and III. Different branch colors represent the three subfamilies. Numbers at the nodes indicate ultrafast bootstrap support values.

To investigate variation among *GPAT* subfamilies across soybean accessions and clarify their phylogenetic relationships, we performed a phylogenetic analysis using 1,329 *GPAT* proteins identified from 29 soybean genomes together with reference *GPAT* proteins from *A. thaliana*. Based on the topology of the phylogenetic tree, the *GPAT* family was divided into three subfamilies, designated I, II, and III. The soybean *GPAT* genes were unevenly distributed among the three subfamilies. Subfamily III contained 645 members and accounted for 48.53% of all soybean *GPAT* genes, making it the largest subfamily (**Figure 2B**; **Supplementary Table S3**). Subfamily II contained 625 members and accounted for 47.03%, whereas subfamily I contained only 59 members and represented 4.44% of the total (**Figure 2B**; **Supplementary Table S3**). Gene number also varied among accessions within each subfamily. Subfamily I contained two to three members per accession. 28 accessions contained two members, while SoyW03 contained three. Subfamily II contained 20 to 23 members per accession. SoyL05 contained the highest number with 23 members, whereas SoyC03, SoyC07, SoyL01, and SoyW03 each contained 20. Subfamily III contained 19 to 23 members per accession (**Figure 2B**; **Supplementary Table S3**). SoyC01 contained the fewest members with 19, while 18 accessions contained 23 members, including W05. A similar subfamily composition was observed across the three soybean germplasm groups. Cultivars contained an average of 2.00, 21.44, and 22.31 genes in subfamilies I, II, and III, respectively. The corresponding averages were 2.00, 21.78, and 21.78 in landraces and 2.25, 21.50, and 23.00 in wild soybeans (**Figure 2B**; **Supplementary Table S3**). Subfamily III was slightly more abundant in wild soybeans, whereas subfamily II showed similar gene numbers across the three groups. Overall, subfamilies II and III comprised the majority of the soybean *GPAT* family. In contrast, subfamily I was much smaller and showed little variation in absolute gene number among accessions.

### Pangenome-wide classification of core and dispensable *GPAT* genes in soybean

To further characterize the conservation and variation of the soybean *GPAT* gene family across different accessions, we used OrthoFinder to classify 1,329 soybean *GPAT* genes into orthologous groups. A total of 1,317 genes were assigned to 47 orthologous groups, accounting for 99.10% of all *GPAT* genes, while 12 genes remained unassigned (**Supplementary Table S3**). Based on their frequencies across the 29 soybean genomes, the orthologous groups were classified as core or dispensable. Orthologous groups present in all 29 genomes were defined as core, whereas those absent from at least one genome were defined as dispensable. The dispensable groups were further divided into softcore, shell, and specific categories. Softcore groups were present in more than 90% but fewer than 100% of the genomes. Shell groups were present in 10% to 90% of the genomes, whereas specific groups were present in fewer than 10% of the genomes. Among the 47 orthologous groups, 19 were classified as core, accounting for 40.43% of the total. The remaining 28 groups were classified as dispensable and accounted for 59.57% (**Supplementary Table S3**). Among the dispensable groups, 22 were softcore, five were shell, and one was specific. These categories accounted for 46.81%, 10.64%, and 2.13% of all orthologous groups, respectively. At the gene level, 603 of the 1,317 assigned *GPAT* genes belonged to core groups, accounting for 45.79%. The remaining 714 genes belonged to dispensable groups and accounted for 54.21% (**Figure 3A**; **Supplementary Table S3**). Among these dispensable genes, 632 were softcore, 80 were shell, and only two were specific. The predominance of softcore genes indicates that most dispensable *GPAT* orthologous groups were still widely distributed across soybean accessions. In contrast, low frequency variation was restricted to a small number of orthologous groups. The only specific orthologous group, OG0000046, was detected in SoyL03 and SoyL04.

**Figure 3:**
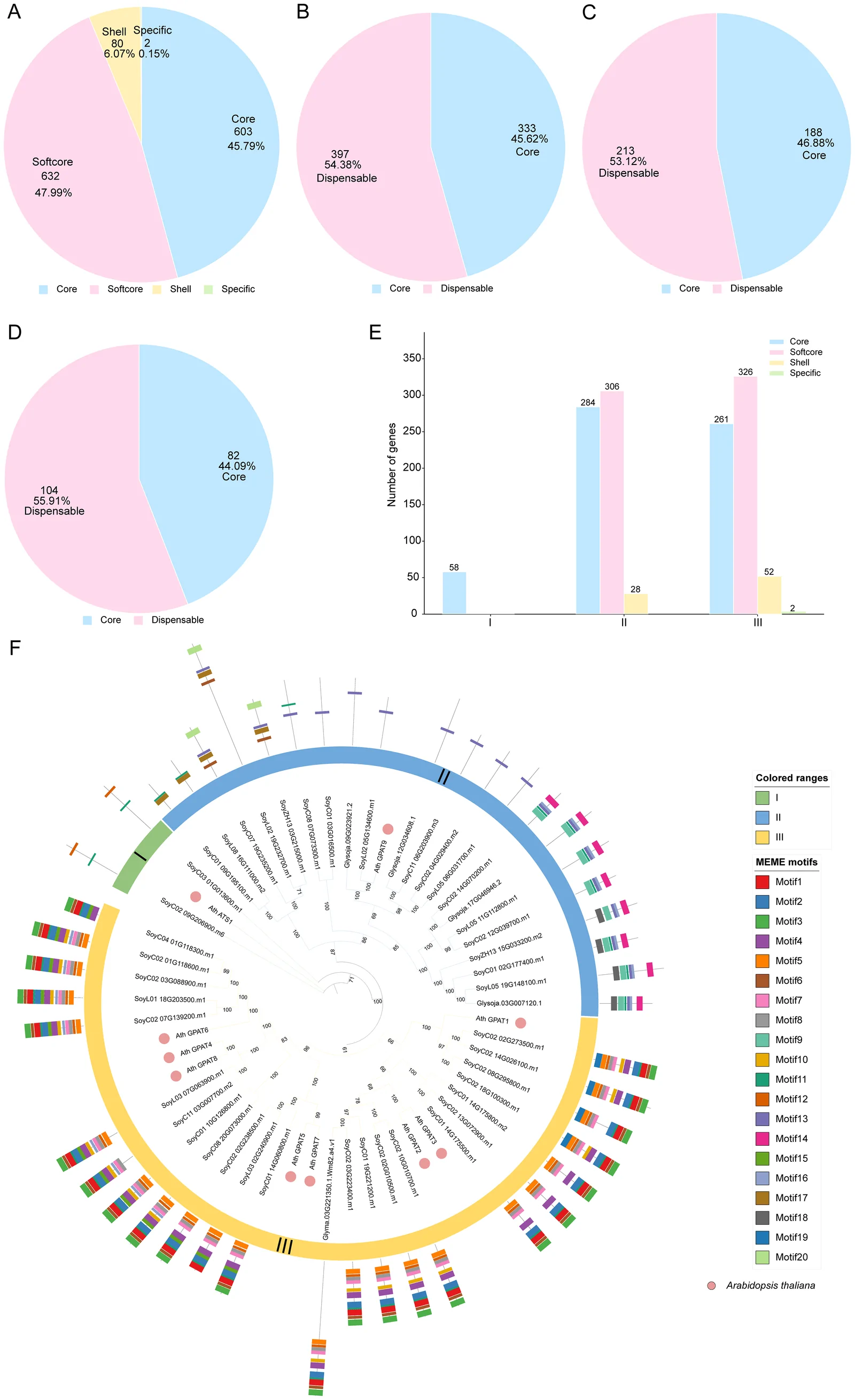
Pangenome composition, subfamily distribution, and conserved motif architecture of soybean *GPAT* orthogroups. **(A)** Composition of core, softcore, shell, and specific *GPAT* genes across 29 soybean accessions. **(B)** Proportions of core and dispensable *GPAT* genes in cultivars. **(C)** Proportions of core and dispensable *GPAT* genes in landraces. **(D)** Proportions of core and dispensable *GPAT* genes in wild soybeans. **(E)** Distribution of core, softcore, shell, and specific *GPAT* genes among subfamilies I, II, and III. Numbers indicate the number of genes in each category. **(F)** Phylogenetic relationships and conserved motif composition of representative soybean *GPAT* orthogroups. For each orthogroup, the longest protein sequence was selected as the representative for phylogenetic analysis.

We further examined whether the distribution of core and dispensable genes differed among cultivars, landraces, and wild soybeans. In cultivars, 45.62% of the assigned *GPAT* genes were core and 54.38% were dispensable (**Figure 3B**; **Supplementary Table S3**). The corresponding proportions were 46.88% and 53.12% in landraces and 44.09% and 55.91% in wild soybeans (**Figures 3C, D**; **Supplementary Table S3**). A chi square test showed no significant association between germplasm group and core or dispensable status (χ^2^ = 0.42, df = 2, P = 0.811). Consistently, the proportion of dispensable genes per accession did not differ significantly among the three germplasm groups based on a Kruskal Wallis test (H = 5.33, P = 0.070). These results indicate that the overall composition of the GPAT gene family remained similar among cultivars, landraces, and wild soybeans. The soybean *GPAT* family therefore shows substantial conservation across accessions, while a limited number of shell and specific orthologous groups contribute to intraspecific variation.

Further comparison among the three *GPAT* subfamilies revealed clear differences in the distribution of core and dispensable genes. Subfamily I contained 59 genes, of which 58 were assigned to orthologous groups and one remained unassigned. All 58 assigned genes were classified as core, and no dispensable genes were detected in this subfamily. The proportion of core genes therefore reached 100%, which was 2.18 fold higher than the overall proportion of 45.79% among all assigned *GPAT* genes (**Figure 3E**; **Supplementary Table S3**). Subfamily II contained 625 genes, with 618 assigned to orthologous groups. Among these genes, 284 were core and 334 were dispensable. Core genes accounted for 45.95% of the assigned genes in this subfamily, which was almost identical to the overall family average. Subfamily III contained 645 genes, of which 641 were assigned to orthologous groups (**Figure 3E**; **Supplementary Table S3**). This subfamily contained 261 core and 380 dispensable genes. Core genes accounted for 40.72% of the assigned genes, whereas dispensable genes accounted for 59.28% (**Figure 3E**; **Supplementary Table S3**). A chi square test showed a significant association between *GPAT* subfamily membership and core or dispensable status, with χ^2^ = 75.32, df = 2, P = 4.42 × 10^-17^, and Cramer’s V = 0.239. Fisher’s exact tests were then used to compare each subfamily with the remaining subfamilies. Subfamily I showed a strong enrichment of core genes. The Fisher’s exact test yielded a P value of 4.42 × 10^-21^ and an adjusted P value of 1.33 × 10^-20^. The odds ratio was infinite because no dispensable genes were detected in this subfamily. In contrast, the proportion of core genes in subfamily II did not differ significantly from the background, with an odds ratio of 1.01 and a P value of 0.912. Subfamily III showed a significant depletion of core genes, with an odds ratio of 0.67 and a P value of 3.95 × 10^-4^. These results indicate distinct patterns of gene conservation among the three *GPAT* subfamilies. Subfamily I showed the highest level of conservation, whereas subfamily III contained a greater proportion of variable genes across soybean accessions.

To further examine the evolutionary relationships and conserved sequence features among *GPAT* orthogroups, the longest protein sequence from each of the 47 orthogroups was selected as a representative for phylogenetic and conserved motif analyses. MEME analysis identified 20 conserved motifs, whose distributions showed clear subfamily-specific patterns (**Figure 3F**). In subfamily III, Motifs 1, 2, and 11 were present in all 24 representative proteins, while Motifs 3, 4, 6, 7, and 8 occurred in 23 of the 24 representatives, and Motifs 5 and 12 were detected in 22, indicating a highly conserved motif architecture within this subfamily. By contrast, subfamily II showed a simpler and more variable motif composition. Motif 13 was the predominant motif and was present in 19 of 21 representatives, whereas Motif 11 occurred in 10 representatives and Motifs 9, 14, and 16 were each detected in eight representatives. The two subfamily I representatives shared only Motifs 11 and 12. Core and dispensable orthogroups within the same subfamily generally exhibited similar major motif compositions; for example, both core and dispensable members of subfamily III consistently retained Motifs 1, 2, and 11 (**Figure 3F**; **Supplementary Table S3**). Overall, the conserved motif patterns were largely consistent with the phylogenetic classification and further revealed pronounced structural differentiation among the three soybean GPAT subfamilies.

### Physicochemical properties of soybean GPAT proteins

Analysis of the physicochemical properties of 1,329 GPAT proteins identified from 29 soybean genomes revealed considerable variation among family members. Protein lengths ranged from 89 to 1,134 amino acids, with a mean of 449.24 and a median of 470 amino acids (**Figures 4A, G**; **Supplementary Table S4**). Molecular weights ranged from 10.24 to 127.75 kDa, with an average of 50.58 kDa (**Figures 4B, H**; **Supplementary Table S4**). The theoretical isoelectric point ranged from 5.07 to 10.30, with a mean of 8.95. A total of 1,250 proteins had a theoretical isoelectric point above 7, accounting for 94.06% of all GPAT proteins (**Figures 4C, I**; **Supplementary Table S4**). This result indicates that most soybean GPAT proteins are basic. The instability index ranged from 30.21 to 67.54, with an average of 42.08. Among these proteins, 786 had an instability index above 40, representing 59.14% of the total (**Figures 4D, J**; **Supplementary Table S4**). This suggests that more than half of the soybean GPAT proteins are predicted to be unstable. The aliphatic index ranged from 73.77 to 112.97, with an average of 96.50 (**Figures 4E, K**; **Supplementary Table S4**). These generally high values indicate a high proportion of aliphatic amino acids in the GPAT proteins. The grand average of hydropathicity ranged from −0.425 to 0.491, with a mean of 0.072. In total, 941 proteins showed positive values, accounting for 70.81% of the family (**Figures 4F, L**; **Supplementary Table S4**). This pattern indicates a mild overall tendency toward hydrophobicity among soybean GPAT proteins.

**Figure 4:**
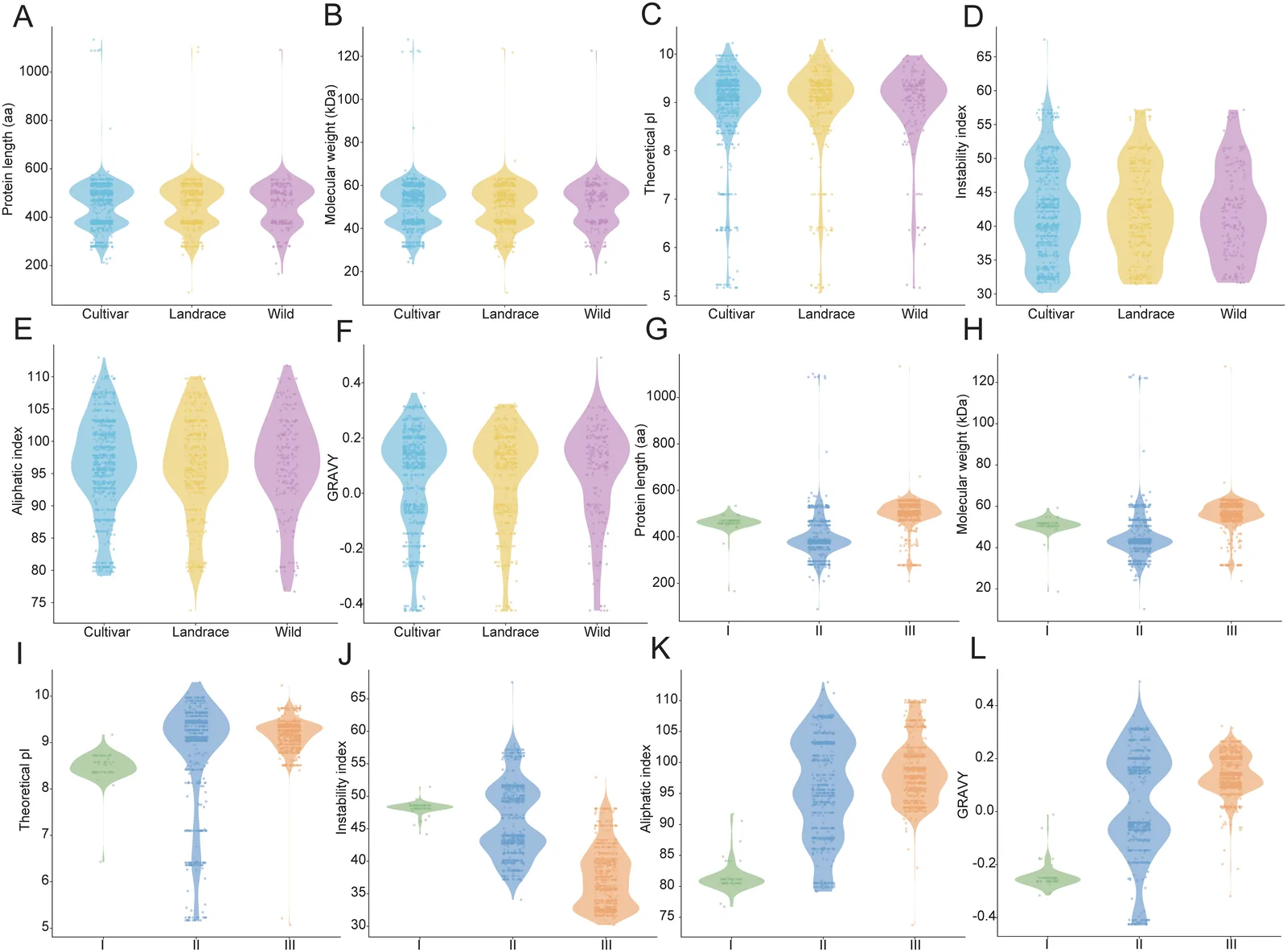
Physicochemical properties of soybean GPAT proteins across germplasm groups and subfamilies. **(A–F)** Comparison of GPAT protein physicochemical properties among cultivars, landraces, and wild soybeans. The analyzed properties include protein length **(A)**, molecular weight **(B)**, theoretical isoelectric point **(C)**, instability index **(D)**, aliphatic index **(E)**, and grand average of hydropathicity **(F)**. **(G–L)** Comparison of the same physicochemical properties among GPAT subfamilies I, II, and III, including protein length **(G)**, molecular weight **(H)**, theoretical isoelectric point **(I)**, instability index **(J)**, aliphatic index **(K)**, and grand average of hydropathicity **(L)**. Violin plots show the distribution of each property, and individual points represent GPAT proteins. Different colors indicate different germplasm groups or *GPAT* subfamilies.

Analysis of GPAT protein properties among cultivars, landraces, and wild soybeans revealed highly similar physicochemical profiles across the three germplasm groups. The mean protein lengths were 449.40, 447.22, and 453.05 amino acids in cultivars, landraces, and wild soybeans, respectively (**Figure 4A**; **Supplementary Table S4**). Their average molecular weights were also similar, at 50.59, 50.35, and 51.00 kDa, respectively (**Figure 4B**; **Supplementary Table S4**). The mean theoretical isoelectric point was approximately 8.95 in all three groups. Basic proteins with a theoretical isoelectric point above 7 accounted for 94.40% of cultivar GPATs, 93.41% of landrace GPATs, and 94.12% of wild soybean GPATs (**Figure 4C**; **Supplementary Table S4**). The mean instability indices were 42.11, 42.12, and 41.89, respectively. Approximately 59% of GPAT proteins in each group had an instability index above 40 (**Figure 4D**; **Supplementary Table S4**). The mean aliphatic indices were also nearly identical, with values of 96.50 in cultivars, 96.53 in landraces, and 96.44 in wild soybeans (**Figure 4E**; **Supplementary Table S4**). Similarly, the mean grand average of hydropathicity values were 0.072, 0.073, and 0.074, respectively. Positive hydropathicity values were observed in 70.36% of cultivar GPATs, 71.46% of landrace GPATs, and 71.12% of wild soybean GPATs (**Figure 4F**; **Supplementary Table S4**). Kruskal–Wallis tests detected no significant differences among the three groups for protein length, molecular weight, theoretical isoelectric point, instability index, aliphatic index, or hydropathicity, with all P values greater than 0.66 (**Figures 4A–F**; **Supplementary Table S4**). These results indicate that the physicochemical properties of GPAT proteins are highly conserved among cultivated, landrace, and wild soybean germplasm.

Further comparison among the three *GPAT* subfamilies revealed pronounced differences in their physicochemical properties. The average protein lengths of subfamilies I, II, and III were 459.49, 397.60, and 498.34 amino acids, respectively (**Figure 4G**; **Supplementary Table S4**). Their average molecular weights were 50.69, 45.11, and 55.87 kDa, respectively (**Figure 4H**; **Supplementary Table S4**). Proteins in subfamily III were generally longer and had higher molecular weights, whereas those in subfamily II were shorter. Kruskal–Wallis tests detected highly significant differences among the three subfamilies in both protein length and molecular weight, with P values of 2.48 × 10^-120^ and 1.96 × 10^-111^, respectively (**Figures 4G, H**; **Supplementary Table S4**). The mean theoretical isoelectric points were 8.47 in subfamily I, 8.77 in subfamily II, and 9.17 in subfamily III. The difference among the three subfamilies was also highly significant, with a P value of 5.34 × 10^-21^ (**Figure 4I**; **Supplementary Table S4**). Clear differences were also observed in protein stability. The mean instability indices were 48.21, 46.18, and 37.55 in subfamilies I, II, and III, respectively. All proteins in subfamily I had instability indices above 40. The corresponding proportions were 86.40% in subfamily II and 28.99% in subfamily III. The instability index differed significantly among the three subfamilies, with a P value of 5.97 × 10^-137^ (**Figure 4J**; **Supplementary Table S4**). The mean aliphatic indices were 81.45, 95.92, and 98.45 in subfamilies I, II, and III, respectively (**Figure 4K**; **Supplementary Table S4**). Their mean grand average of hydropathicity values were −0.245, 0.032, and 0.141, respectively. All proteins in subfamily I had negative hydropathicity values, indicating a strong hydrophilic tendency. In contrast, 49.76% of subfamily II proteins and 97.67% of subfamily III proteins had positive values. Subfamily III therefore showed the strongest tendency toward hydrophobicity. Significant differences were also detected in aliphatic index and hydropathicity, with P values of 5.30 × 10^-37^ and 4.85 × 10^-42^, respectively (**Figures 4K, L**; **Supplementary Table S4**). Overall, these results reveal distinct physicochemical characteristics among the three soybean *GPAT* subfamilies. Subfamily I was characterized by greater predicted instability and hydrophilicity, whereas subfamily III showed greater predicted stability and hydrophobicity.

Further comparison of the 1,317 GPAT proteins assigned to orthologous groups revealed differences in several physicochemical properties between proteins encoded by core and dispensable genes. The 603 core GPAT proteins had an average length of 446.39 amino acids and an average molecular weight of 50.19 kDa (**Supplementary Table S5**). The corresponding values for the 714 dispensable proteins were 453.61 amino acids and 51.13 kDa. Mann Whitney U tests followed by Benjamini Hochberg correction showed no significant differences in protein length or molecular weight, with adjusted P values of 0.890 and 0.075, respectively (**Supplementary Table S5**). In contrast, core proteins had a higher mean theoretical isoelectric point than dispensable proteins, with values of 9.12 and 8.82, respectively. This difference was highly significant, with an adjusted P value of 9.24 × 10^-9^. In addition, 99.83% of core proteins had theoretical isoelectric points above 7, compared with 89.36% of dispensable proteins (**Supplementary Table S5**). Core proteins also had a higher mean instability index than dispensable proteins, with values of 42.76 and 41.49, respectively. A total of 64.68% of core proteins had instability indices above 40, compared with 54.48% of dispensable proteins (**Supplementary Table S5**). The difference in instability index was significant, with an adjusted P value of 0.003. The mean aliphatic indices were 96.30 for core proteins and 96.74 for dispensable proteins. Their mean grand average of hydropathicity values were 0.063 and 0.082, respectively (**Supplementary Table S5**). No significant differences were detected in either aliphatic index or hydropathicity, with adjusted P values of 0.890 and 0.836, respectively (**Supplementary Table S5**). All in all, core GPAT proteins tended to have higher isoelectric points and higher predicted instability than dispensable proteins. Other physicochemical properties remained largely similar between the two groups.

### Analysis of CNVs and gene duplication types in soybean *GPAT* families

Analysis of duplication types among the 1,329 soybean *GPAT* genes showed that WGD or segmental duplication was the predominant mode of family expansion. A total of 1,148 genes were classified as WGD or segmental duplicates, accounting for 86.38% of all *GPAT* genes. Dispersed duplication was the second most common type, with 127 genes representing 9.56% of the total. Proximal duplication accounted for 52 genes, or 3.91%. Only one tandem duplicate and one singleton were identified, and each represented 0.08% of the family (**Figure 5A**; **Supplementary Table S6**). Overall, these results indicate that whole genome duplication and large-scale segmental duplication played the major role in the expansion of the soybean *GPAT* gene family. Further comparison among the 29 soybean accessions showed that WGD or segmental duplication was the dominant duplication type in every accession. The number of WGD or segmental duplicated *GPAT* genes ranged from 35 to 41 and accounted for 82.61% to 90.70% of the *GPAT* genes in individual accessions. SoyC12 showed the highest proportion at 90.70%, followed by Wm82 at 89.13%. SoyC03 and SoyL04 both showed proportions of 88.89%. In contrast, SoyC13 and SoyW03 showed the lowest proportions at 82.61%, followed by SoyC10 at 82.98% and SoyC01 at 83.33% (**Figure 5A**; **Supplementary Table S6**). Despite this variation, more than 80% of *GPAT* genes in every accession were classified as WGD or segmental duplicates. This consistent pattern further supports the major contribution of whole genome duplication and segmental duplication to *GPAT* family retention and expansion across soybean genomes.

**Figure 5:**
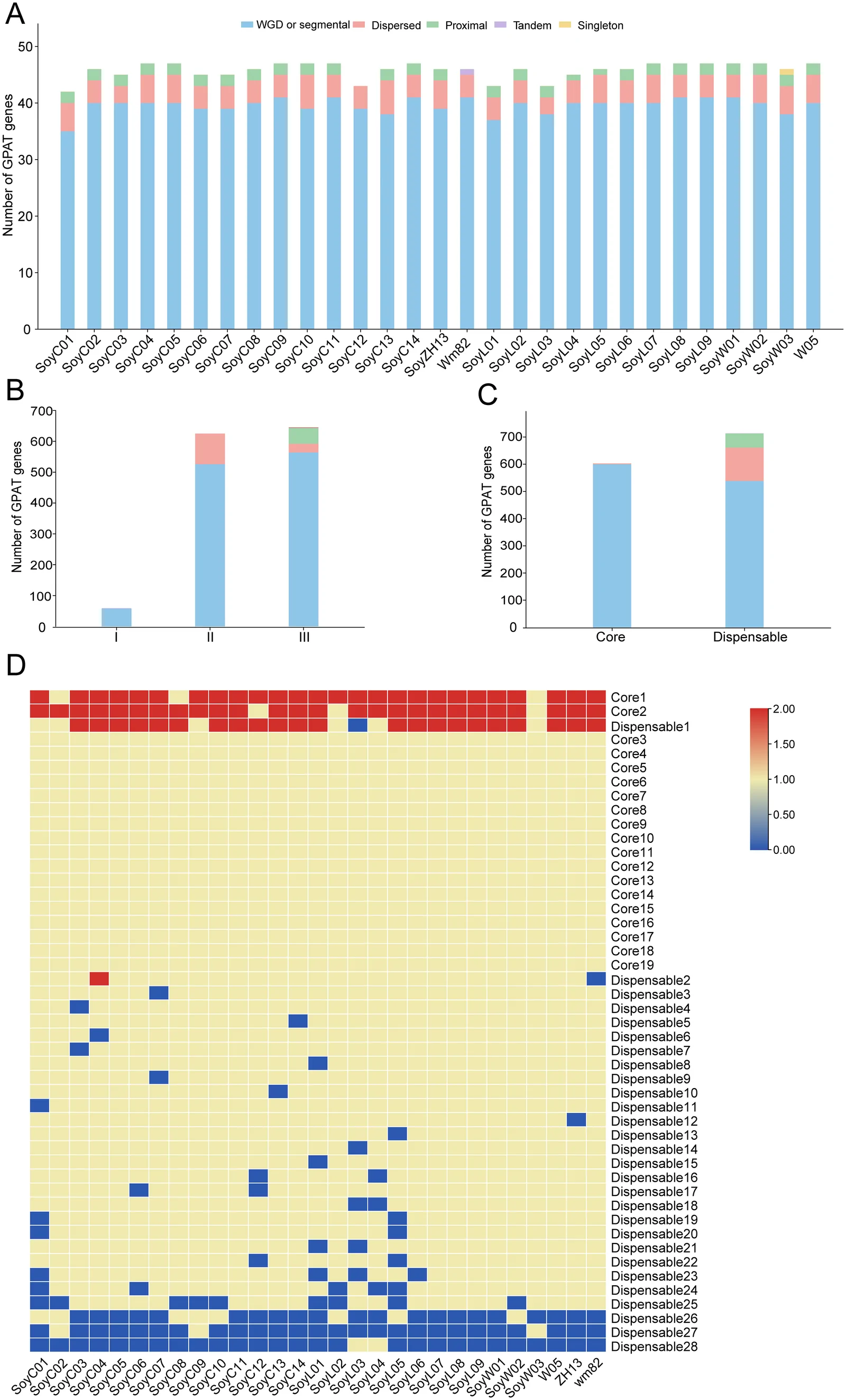
Duplication patterns and copy number variation of the soybean *GPAT* gene family. **(A)** Distribution of *GPAT* gene duplication types across 29 soybean accessions. Stacked bars show the numbers of genes classified as WGD or segmental, dispersed, proximal, tandem, and singleton. **(B)** Distribution of duplication types among *GPAT* subfamilies I, II, and III. **(C)** Comparison of duplication type composition between core and dispensable *GPAT* genes. **(D)** Copy number variation of 47 *GPAT* orthogroups across the 29 soybean accessions.

The composition of duplication types differed markedly among the three *GPAT* subfamilies. Of the 59 genes in subfamily I, 58 were classified as WGD or segmental duplicates, accounting for 98.31%, while the remaining gene was a singleton. No dispersed, proximal, or tandem duplicates were detected in this subfamily. Subfamily II contained 625 genes, of which 526 were WGD or segmental duplicates and 99 were dispersed duplicates, representing 84.16% and 15.84%, respectively. No proximal or tandem duplicates were detected in subfamily II. Among the 645 genes in subfamily III, 564 were WGD or segmental duplicates, accounting for 87.44%. In addition, 52 genes were proximal duplicates and 28 were dispersed duplicates, accounting for 8.06% and 4.34%, respectively. The only tandem duplicate identified in the entire *GPAT* family also belonged to subfamily III (**Figure 5B**; **Supplementary Table S6**). A chi-square test detected a highly significant association between subfamily membership and duplication type, with χ^2^ = 128.96, df = 8, P = 4.64 × 10^-24^, and Cramer’s V = 0.220 (**Supplementary Table S6**). Because singleton and tandem duplicates were rare, Fisher’s exact tests were further used to evaluate enrichment of the major duplication types. WGD or segmental duplicates were significantly enriched in subfamily I, with an odds ratio of 9.58 and an adjusted P value of 0.0087. Dispersed duplicates were significantly enriched in subfamily II, with an odds ratio of 4.54 and an adjusted P value of 5.72 × 10^-13^. Proximal duplicates were strongly enriched in subfamily III, with an adjusted P value of 2.35 × 10^-16^ (**Supplementary Table S6**). Overall, WGD or segmental duplication was the major source of *GPAT* genes in all three subfamilies. However, the relative contribution of other duplication mechanisms differed substantially among subfamilies. These results suggest distinct patterns of gene retention and expansion during the evolution of the soybean *GPAT* subfamilies.

Comparison of the 1,317 *GPAT* genes assigned to orthologous groups revealed pronounced differences in duplication type between core and dispensable genes. Among the 603 core genes, 600 were classified as WGD or segmental duplicates, accounting for 99.50%, while only three genes were classified as dispersed duplicates, representing 0.50%. No proximal or tandem duplicates were detected among core genes. In contrast, the 714 dispensable genes showed a more diverse duplication pattern. A total of 539 genes were WGD or segmental duplicates, accounting for 75.49%, while 122 were dispersed duplicates, representing 17.09%. Proximal duplication accounted for 52 genes, or 7.28%, and one tandem duplicate was also identified (**Figure 5C**; **Supplementary Table S6**). An overall association test showed a highly significant difference in duplication type composition between core and dispensable genes, with χ^2^ = 161.35, df = 3, P = 9.39 × 10^-35^, and Cramer’s V = 0.350 (**Supplementary Table S6**). These results indicate that core *GPAT* genes are strongly associated with WGD or segmental duplication. In contrast, dispersed and proximal duplication contributed more strongly to the dispensable component of the family. This pattern suggests that genes retained after large-scale duplication events are more likely to remain conserved across soybean accessions, whereas genes generated by other duplication mechanisms contribute more to intraspecific variation in the *GPAT* family.

Among the 47 *GPAT* orthogroups, 17 were classified as single-copy orthogroups because they contained exactly one gene in each of the 29 soybean accessions. These groups accounted for 36.17% of all orthogroups and contained 493 genes. The remaining 30 orthogroups showed copy number variation and were classified as CNV orthogroups, accounting for 63.83% of the total and containing 824 genes. Among the 30 CNV orthogroups, 28 also showed presence and absence variation among accessions. Only OG0000000 (Core1) and OG0000001 (Core2) were retained in all 29 accessions but varied between one and two copies (**Figure 5D**; **Supplementary Table S7**). The duplication type composition differed strongly between single-copy and CNV genes. Among the 493 genes from single-copy orthogroups, 490 were classified as WGD or segmental duplicates, accounting for 99.39%, while only three were dispersed duplicates. No proximal or tandem duplicates were detected in this group. In contrast, the 824 genes from CNV orthogroups showed a more diverse duplication pattern. A total of 649 genes were WGD or segmental duplicates, accounting for 78.76%. Dispersed and proximal duplicates accounted for 122 and 52 genes, representing 14.81% and 6.31%, respectively. One tandem duplicate was also detected (**Supplementary Table S7**). A Fisher–Freeman–Halton exact test was used. The overall association between copy number category and duplication type was highly significant, with P = 1.28 × 10^-33^ and Cramer’s V = 0.292 (**Supplementary Table S7**). Pairwise Fisher exact tests followed by Benjamini–Hochberg correction showed a strong enrichment of WGD or segmental duplicates in single-copy genes, with an odds ratio of 44.04 and an adjusted P value of 3.41 × 10^-34^. Dispersed duplicates were significantly enriched among CNV genes, with an odds ratio of 28.39 and an adjusted P value of 3.56 × 10^-22^. Proximal duplicates were found exclusively among CNV genes and also showed significant enrichment, with an adjusted P value of 3.29 × 10^-11^. The single tandem duplicate did not show significant enrichment (**Supplementary Table S7**). These indicate that single-copy *GPAT* orthogroups are almost entirely composed of genes retained after WGD or segmental duplication. In contrast, CNV orthogroups contain a substantially larger contribution from dispersed and proximal duplication. This pattern suggests that long-term retention of WGD or segmental duplicates contributes to the conserved component of the soybean *GPAT* family, whereas more localized duplication processes contribute more strongly to copy number variation among soybean accessions.

### Selective pressure and evolutionary divergence of the soybean *GPAT* gene family

To evaluate the selective pressures acting on the soybean *GPAT* gene family during intraspecific evolution, we analyzed Ka, Ks, and the Ka to Ks ratio for homologous *GPAT* gene pairs within the same orthologous groups across 29 soybean accessions. A total of 6,494 valid and nonredundant gene pair comparisons were obtained. Overall, the mean and median Ka values were 0.0106 and 0.00117, respectively, while the mean and median Ks values were 0.0236 and 0.00573. The mean Ka to Ks ratio was 0.362, with a median of 0.305. Among the 6,494 gene pairs, 6,107 had a Ka to Ks ratio below 1, accounting for 94.04% of the total. Only 387 pairs had a ratio above 1, representing 5.96% (**Supplementary Table S8**). Most homologous *GPAT* genes have been predominantly subjected to purifying selection during intraspecific divergence. These results indicate that purifying selection predominated during the intraspecific divergence of soybean *GPAT* genes.

Further comparison of selective pressures among cultivars, landraces, and wild soybeans revealed significant differences in Ka, Ks, and the Ka to Ks ratio. Only gene pairs in which both genes belonged to the same germplasm group were included in this comparison. A total of 1,672 cultivar pairs, 491 landrace pairs, and 171 wild soybean pairs were analyzed. The mean Ka values were 0.0140 in cultivars, 0.00739 in landraces, and 0.00778 in wild soybeans. The corresponding mean Ks values were 0.0315, 0.0132, and 0.0205, respectively. The mean Ka to Ks ratios were 0.395 in cultivars, 0.412 in landraces, and 0.225 in wild soybeans. Wild soybean gene pairs therefore showed the lowest mean Ka to Ks ratio among the three groups (**Figures 6A–C**; **Supplementary Table S8**). Kruskal–Wallis tests detected significant differences among the three germplasm groups for Ka, Ks, and the Ka to Ks ratio, with P values of 5.35 × 10^-8^, 9.33 × 10^-4^, and 2.22 × 10^-5^, respectively (**Figures 6A–C**; **Supplementary Table S8**). Pairwise Mann–Whitney U tests followed by Benjamini–Hochberg correction showed significant differences in Ka between cultivars and landraces, cultivars and wild soybeans, and landraces and wild soybeans. The adjusted P values were 1.58 × 10^-3^, 7.55 × 10^-7^, and 9.36 × 10^-4^, respectively. For Ks, significant differences were detected between cultivars and landraces and between cultivars and wild soybeans, with adjusted P values of 0.0199 and 0.00694. The difference between landraces and wild soybeans was not significant, with an adjusted P value of 0.0604. For the Ka to Ks ratio, wild soybeans differed significantly from both cultivars and landraces, with adjusted P values of 1.12 × 10^-5^ and 2.12 × 10^-4^, respectively. No significant difference was detected between cultivars and landraces, with an adjusted P value of 0.494 (**Supplementary Table S8**). These suggest that *GPAT* genes in wild soybeans have experienced stronger purifying selection than those in cultivated and landrace germplasm, whereas cultivars and landraces showed broadly similar selective constraints based on their Ka to Ks ratios.

**Figure 6:**
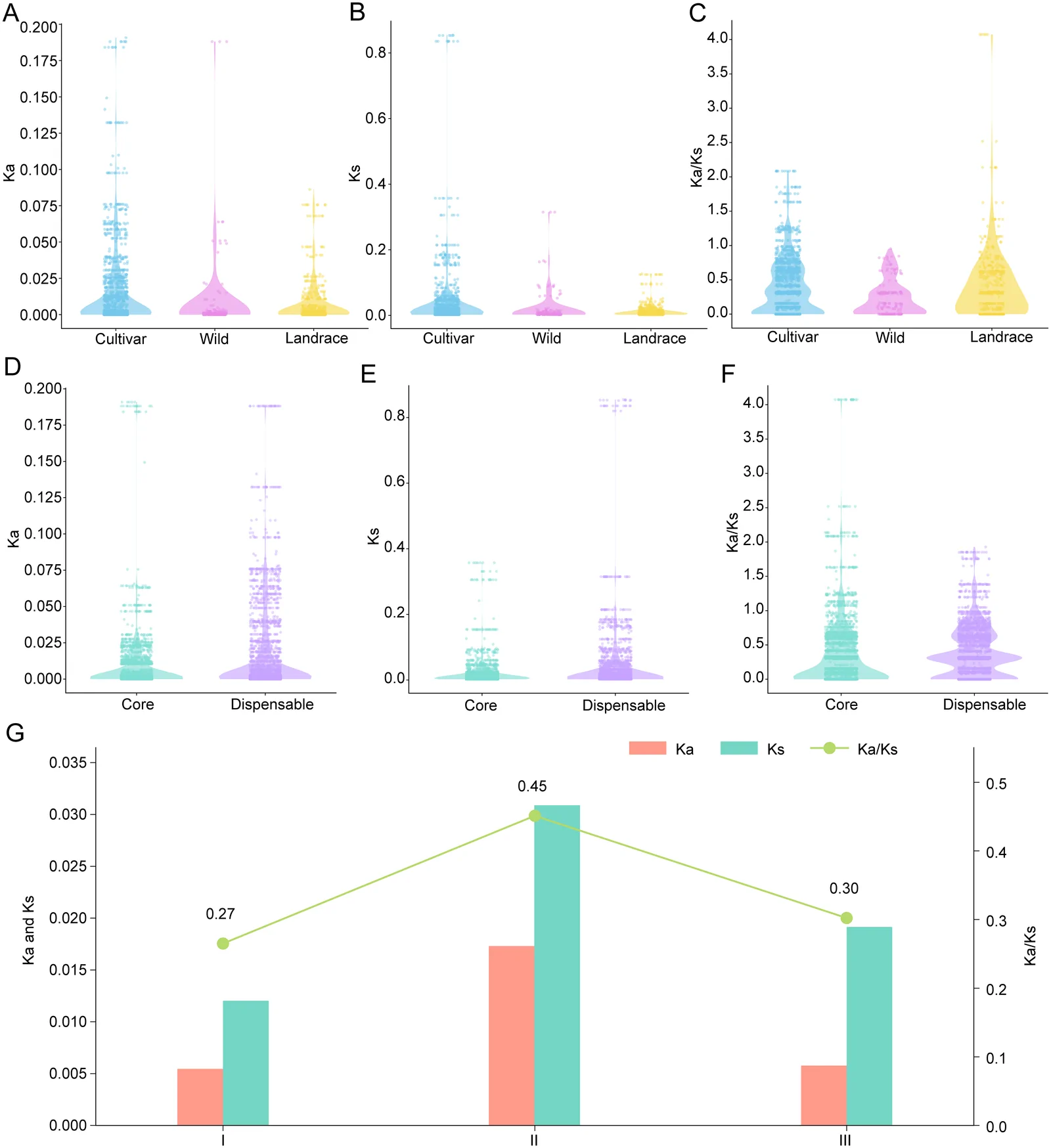
Selective pressure on soybean *GPAT* genes across germplasm groups, gene categories, and subfamilies. **(A–C)** Distribution of Ka **(A)**, Ks **(B)**, and Ka to Ks ratios **(C)** among cultivars, wild soybeans, and landraces. **(D–F)** Distribution of Ka **(D)**, Ks **(E)**, and Ka to Ks ratios **(F)** between core and dispensable *GPAT* gene pairs. Violin plots show the distribution of values, and individual points represent homologous *GPAT* gene pairs. **(G)** Mean Ka, Ks, and Ka to Ks ratios of *GPAT* subfamilies I, II, and III. Bars represent mean Ka and Ks values, while the line represents the mean Ka to Ks ratio. Numbers above the points indicate the mean Ka to Ks ratio for each subfamily.

Core and dispensable *GPAT* genes showed distinct evolutionary patterns. A total of 3,119 homologous gene pairs belonged to core orthologous groups, whereas 3,375 pairs belonged to dispensable orthologous groups. The mean Ka values were 0.00728 for core gene pairs and 0.0137 for dispensable gene pairs, with median values of 0.000934 and 0.00158, respectively. The mean Ks values were 0.0167 and 0.0299, while the corresponding median values were 0.00619 and 0.00525. The mean Ka to Ks ratio was 0.335 for core gene pairs and 0.388 for dispensable gene pairs. Their median values were 0.150 and 0.309, respectively (**Figures 6D–F**; **Supplementary Table S8**). Mann–Whitney U tests followed by Benjamini–Hochberg correction showed a highly significant difference in Ka between the two groups, with an adjusted P value of 4.61 × 10^-34^. A highly significant difference was also detected for the Ka to Ks ratio, with an adjusted P value of 1.23 × 10^-29^. In contrast, Ks did not differ significantly between core and dispensable gene pairs, with an adjusted P value of 0.243 (**Figures 6D–F**; **Supplementary Table S8**). Cliff’s delta values were −0.173 for Ka, 0.017 for Ks, and −0.160 for the Ka to Ks ratio, indicating small effect sizes for the significant differences. Among core gene pairs, 7.44% had a Ka to Ks ratio above 1, compared with 4.59% of dispensable gene pairs. Fisher’s exact test showed that this difference was significant, with an odds ratio of 1.67 and a P value of 1.29 × 10^-6^ (**Supplementary Table S8**). Core gene pairs showed significantly lower median Ka/Ks ratios than dispensable gene pairs, indicating a lower overall Ka/Ks distribution in core genes. However, the proportion of gene pairs with Ka/Ks > 1 was significantly higher in core genes than in dispensable genes (7.44% vs. 4.59%), suggesting heterogeneous evolutionary patterns among core genes, with most remaining relatively conserved while a small subset may have experienced accelerated coding-sequence divergence.

Further comparison among the three *GPAT* subfamilies revealed clear differences in selective pressure. A total of 500, 2,746, and 3,248 homologous gene pairs were assigned to subfamilies I, II, and III, respectively. The mean Ka values were 0.00546, 0.01730, and 0.00577 for subfamilies I, II, and III, respectively. Their mean Ks values were 0.01203, 0.03089, and 0.01914, while the mean Ka to Ks ratios were 0.265, 0.451, and 0.302, respectively. Subfamily II showed the highest mean values for all three measures, whereas subfamily I showed the lowest (**Figure 6G**; **Supplementary Table S8**). Kruskal–Wallis tests detected highly significant differences among the three subfamilies for Ka, Ks, and the Ka to Ks ratio, with P values of 1.04 × 10^-78^, 1.44 × 10^-92^, and 1.23 × 10^-36^, respectively (**Supplementary Table S8**). Pairwise Mann–Whitney U tests followed by Benjamini–Hochberg correction showed significant differences in Ka between subfamilies I and II and between subfamilies II and III. No significant difference was detected between subfamilies I and III. Ks differed significantly among all three pairwise comparisons. For the Ka to Ks ratio, significant differences were detected between subfamilies I and II and between subfamilies II and III, whereas the difference between subfamilies I and III was not significant (**Supplementary Table S8**). In addition, only 0.40% of gene pairs in subfamily I had a Ka to Ks ratio above 1, compared with 8.92% in subfamily II and 4.31% in subfamily III. These proportions differed significantly among the three subfamilies, with χ^2^ = 86.34, df = 2, and P = 1.78 × 10^-19^ (**Supplementary Table S8**). Overall, subfamily I showed the lowest Ka to Ks ratio and the smallest proportion of gene pairs with values above 1, indicating strong purifying selection. Subfamily II showed the highest Ka to Ks ratio and the largest proportion of gene pairs with values above 1, suggesting weaker selective constraint and greater coding sequence divergence. Subfamily III showed an intermediate pattern, although its Ka to Ks ratio did not differ significantly from that of subfamily I.

### *GPAT* pan-transcriptome reveals pronounced tissue specific expression and transcriptional divergence within subfamilies

To characterize the transcriptional features of the soybean *GPAT* gene family at the intraspecific level, we analyzed the expression patterns of 47 *GPAT* pangenes across eight tissues using pan-transcriptome data from 29 soybean accessions. For each pangene, the mean TPM across accessions was transformed using log2(mean TPM + 1) before downstream analysis. Overall, *GPAT* pangenes showed pronounced differences in expression among tissues. Flower showed the highest mean transformed expression value at 2.95, followed by Leaf_bud and Pod at 2.56 and 2.53, respectively. Pod_seed, Leaf, and Stem showed intermediate mean expression values of 2.26, 2.23, and 2.10. Root and Seed showed the lowest overall expression, with mean values of 1.49 and 1.53, respectively (**Figure 7A**; **Supplementary Table S9**). These results indicate substantial transcriptional differentiation of the *GPAT* family among soybean tissues. Many pangenes showed relatively high transcriptional activity in reproductive and developmental tissues, particularly Flower, Leaf_bud, Pod, and Seed. Expression levels also varied markedly among individual pangenes. Core6 showed the highest expression in Flower, with a transformed value of 8.54, whereas its expression values in Root and Seed were only 2.68 and 1.28, respectively. Core7 was highly expressed in Flower, Leaf_bud, Pod_seed, and Pod, with values of 6.97, 6.82, 6.44, and 7.38, respectively. In contrast, its expression was much lower in Root and Seed, with values of 0.89 and 1.04, respectively. Core13 also showed high transcriptional activity in several tissues, reaching 7.05 in Flower, 6.91 in Leaf_bud, and 6.74 in Pod. Among dispensable pangenes, Dispensable23 showed particularly high expression in Flower, Leaf, and Pod, with values of 7.17, 6.98, and 7.31, respectively, whereas its expression in Root was only 1.44. Other dispensable pangenes showed strong tissue-biased patterns. For example, Dispensable1 reached 4.54 in Leaf_bud but showed very low expression in Leaf, Pod, Root, and Seed (**Figure 7A**; **Supplementary Table S9**). These results reveal extensive variation in transcriptional activity among soybean *GPAT* pangenes and suggest substantial functional differentiation across tissues.

**Figure 7:**
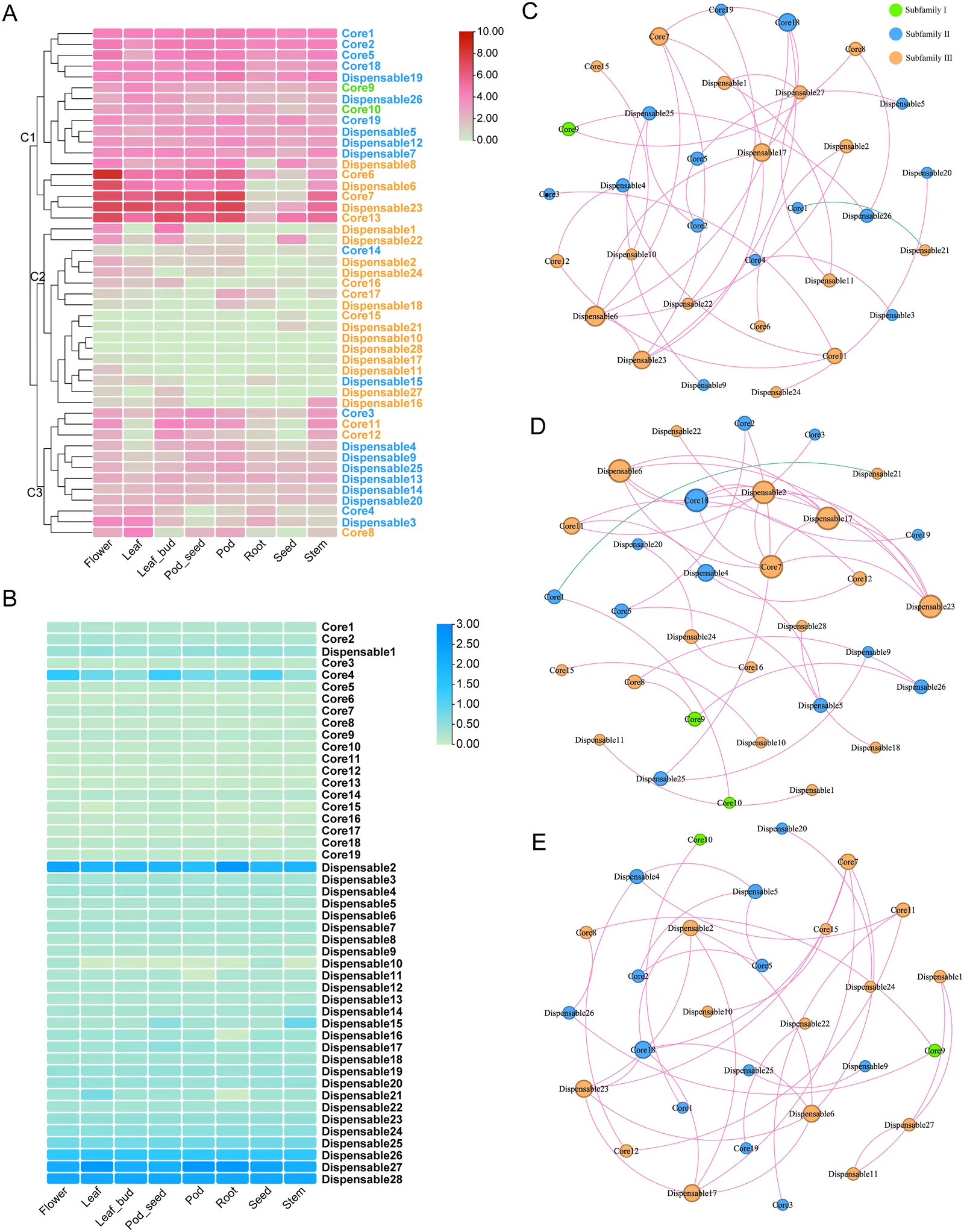
Pan-transcriptome expression patterns and co-expression networks of the soybean *GPAT* gene family. **(A)** Heatmap showing the expression patterns of 47 *GPAT* pangenes across eight soybean tissues. Expression values are represented as log2(mean TPM + 1). Hierarchical clustering separated the *GPAT* pangenes into three major expression clusters, C1, C2, and C3. Pangene labels are colored according to *GPAT* subfamily. **(B)** Heatmap showing the transformed relative standard deviation of *GPAT* pangene expression across the eight tissues. Higher values indicate greater expression variation among soybean accessions. **(C–E)** *GPAT* co-expression networks constructed separately for cultivars **(C)**, landraces **(D)**, and wild soybeans **(E)**. Nodes represent *GPAT* pangenes and are colored according to subfamily. Node size reflects network connectivity. Edges represent significant Pearson correlations between pangene expression profiles. Pink edges indicate positive correlations, while green edges indicate negative correlations.

Based on their expression patterns across the eight tissues, the 47 *GPAT* pangenes were divided into three major expression clusters, C1, C2, and C3. C1 contained 18 pangenes and showed the highest overall transcriptional activity, with a mean expression value of 3.82. Flower and Pod showed the highest mean expression in this cluster, with values of 4.75 and 4.49, respectively. C2 contained 17 pangenes and showed the lowest overall expression, with a mean value of 0.64. C3 contained 12 pangenes and showed an intermediate expression level, with a mean value of 2.00 (**Figure 7A**; **Supplementary Table S9**). The three *GPAT* subfamilies showed distinct but overlapping distributions among the expression clusters. Both pangenes from subfamily I were assigned to C1. Subfamily II contributed 10, 2, and 9 pangenes to C1, C2, and C3, respectively, whereas subfamily III contributed 6, 15, and 3 pangenes (**Figure 7A**; **Supplementary Table S9**). This distribution indicates substantial transcriptional divergence within *GPAT* subfamilies and similar expression patterns among some phylogenetically distinct members. We further examined expression variation among the 29 soybean accessions using the transformed relative SD of each pangene. Expression level was significantly negatively correlated with relative SD across all pangene and tissue combinations, with a Spearman correlation coefficient of ρ = −0.190 and P = 2.06 × 10^-4^. However, the correlation was not significant when expression and relative SD were averaged across the eight tissues for each pangene, with ρ = −0.213 and P = 0.151 (**Figure 7B**; **Supplementary Table S10**). Core pangenes showed considerably lower expression variation than dispensable pangenes. Their mean transformed relative SD values were 0.17 and 0.60, respectively. This difference was significant according to a Mann–Whitney U test, with P = 2.17 × 10^-6^ (**Figure 7B**; **Supplementary Table S10**). These results suggest that highly conserved *GPAT* pangenes generally maintain more stable transcription across soybean accessions, whereas dispensable members show greater transcriptional variability.

We mapped transcriptomic expression data from different tissues of cultivated, landrace, and wild soybeans to their corresponding orthologous groups (**Supplementary Table S11**). We then calculated Pearson correlation coefficients between *GPAT* pangenes and constructed separate co-expression networks for the three germplasm groups to identify potential expression hubs within the *GPAT* family (**Figures 7C–E**; **Supplementary Table S12**). Comparison of the *GPAT* co-expression networks among cultivars, landraces, and wild soybeans revealed a highly conserved network structure. The cultivar network contained 31 edges connecting 32 nodes, while the landrace network contained 35 edges and 32 nodes. The wild soybean network contained 33 edges but only 29 nodes. Network density was therefore highest in wild soybeans at 0.081, followed by landraces at 0.071 and cultivars at 0.063 (**Figures 7C–E**; **Supplementary Table S12**). This suggests a more compact *GPAT* co-expression network in wild soybeans. A total of 27 nodes and 25 edges were shared among all three networks, indicating a strongly conserved co-expression backbone. Landraces and wild soybeans shared 30 edges, whereas cultivars shared 27 edges with each of the other two groups (**Figures 7C–E**; **Supplementary Table S12**). Most correlations were positive and showed similar strengths among the three groups. The mean absolute PCC values were 0.974 in cultivars, 0.972 in landraces, and 0.973 in wild soybeans. Only one negative correlation was detected in both cultivars and landraces, between Core1 and Dispensable21, whereas all correlations in wild soybeans were positive (**Figures 7C–E**; **Supplementary Table S12**). Subfamily III contributed the largest number of nodes in all three networks, with 17 in cultivars, 18 in landraces, and 15 in wild soybeans. Subfamily II contributed 14, 12, and 12 nodes, respectively, while subfamily I contributed only 1, 2, and 2 nodes (**Figures 7C–E**; **Supplementary Table S12**). The distribution of edges among subfamilies was also highly similar. Interactions between two subfamily III members were the most abundant, with 13, 15, and 14 edges in cultivars, landraces, and wild soybeans, respectively. Interactions between subfamilies II and III were the second most common, with 11, 12, and 11 edges (**Figures 7C–E**; **Supplementary Table S12**). Several highly connected nodes were conserved across the three networks. Core7, Core18, Dispensable6, Dispensable17, and Dispensable23 consistently showed high connectivity, suggesting that they form a stable central module of the soybean *GPAT* co-expression network (**Figures 7C–E**; **Supplementary Table S12**). In contrast, only a small number of group-specific connections were observed, including two in cultivars, three in landraces, and one in wild soybeans (**Figures 7C–E**; **Supplementary Table S12**). Overall, these results indicate that the major structure of the *GPAT* co-expression network is conserved across soybean germplasm groups, while limited changes in individual nodes and edges contribute to germplasm-specific transcriptional organization.

## Discussion

Our survey across 1,011 plant species revealed the widespread distribution of *GPAT* genes and substantial variation in family size among plant lineages (**Figure 1**; **Supplementary Table S1**). In soybean, the 1,329 *GPAT* genes identified across 29 accessions were classified into three subfamilies, with broadly conserved family size and subfamily composition among cultivars, landraces, and wild soybeans (**Figures 2A, B**; **Supplementary Tables S2, 3**). Despite this overall conservation, orthogroup-level analyses uncovered presence–absence and copy-number variation (**Figures 3A–D**, **5D**; **Supplementary Tables S3**, **S7**), together with subfamily-specific differences in gene conservation, conserved motif architecture, and protein properties (**Figures 3E, F**, **4G–L**; **Supplementary Tables S4, S5**). Whole-genome or segmental duplication dominated family expansion (**Figures 5A–C**; **Supplementary Table S6**), and most homologous gene pairs showed evidence of purifying selection, while core genes showed a lower overall Ka/Ks distribution than dispensable genes (**Figures 6D–F**; **Supplementary Table S8**). Pan-transcriptome analyses further revealed tissue-dependent expression and transcriptional divergence within subfamilies (**Figure 7A**; **Supplementary Table S9**), while core pangenes exhibited greater expression stability across accessions (**Figure 7B**; **Supplementary Table S10**) and the major co-expression network structure remained conserved among germplasm groups (**Figures 7C–E**; **Supplementary Tables S11, 12**). Together, these findings highlight the value of integrating pangenome-based gene-family analysis with pan-transcriptome profiling to distinguish conserved and variable components and resolve intraspecific genetic and transcriptional diversity that cannot be fully captured by a single reference genome. This framework provides candidate GPAT genes and testable hypotheses for future functional studies of soybean lipid traits and stress tolerance.

### Gene duplication and selective constraints shape conserved and variable components of the soybean *GPAT* family

Our results suggest that the conserved and variable components of the soybean *GPAT* family differ in both duplication history and coding-sequence constraint (**Figures 5C**, **6D–F**; **Supplementary Tables S6**, **S8**). WGD or segmental duplicates accounted for 86.38% of all *GPAT* genes and 99.50% of core members, indicating a strong association between this duplication category and widespread retention across accessions (**Figures 5A, C**; **Supplementary Table S6**). The predominance of these duplicates is consistent with the palaeopolyploid history of soybean, whose genome retains numerous duplicated genes following successive genome duplication events (Schmutz et al., 2010; Roulin et al., 2013). Dispersed and proximal duplicates were more frequent among dispensable members and genes belonging to CNV orthogroups, suggesting that different duplication histories are associated with distinct patterns of gene-content conservation (**Figures 5C, D**; **Supplementary Table S6**). A comparable pattern has been reported for barley bHLHs, with WGD or segmental duplication contributing predominantly to core and softcore members and smaller-scale duplication contributing more strongly to dispensable members (Tong et al., 2025b). Selection-pressure analyses provided complementary evidence: 94.04% of homologous *GPAT* gene pairs had Ka/Ks ratios below 1 (**Supplementary Table S8**), and core gene pairs exhibited a lower median ratio than dispensable pairs, suggesting stronger overall coding-sequence constraints on broadly retained members (**Figures 6D–F**; **Supplementary Table S8**). The higher proportion of Ka/Ks > 1 pairs among core genes does not necessarily contradict their lower overall Ka/Ks distribution. Rather, it suggests heterogeneous evolutionary dynamics within core GPAT genes: most core genes remain relatively conserved, whereas a small subset may have undergone accelerated amino-acid sequence divergence. Therefore, differences between core and dispensable genes should be interpreted from the overall Ka/Ks distribution together with the proportion of gene pairs exceeding 1. These patterns may reflect the requirement to preserve established lipid-metabolic functions. For example, Arabidopsis *GPAT9* combines strong evolutionary conservation with experimentally demonstrated roles in gametophyte viability and seed oil accumulation (Shockey et al., 2016). Other plant GPAT lineages possess distinct substrate preferences and enzymatic activities associated with cutin and suberin biosynthesis, illustrating the range of lipid-related functions potentially subject to selective constraint (Yang et al., 2012). Such functional requirements may help explain the broad retention and stronger sequence constraints of core soybean *GPAT*s, while the greater representation of dispersed and proximal duplicates among dispensable members points to a complementary contribution to intraspecific gene-content variation.

### *GPAT* subfamilies exhibit distinct patterns of gene-content and coding-sequence divergence

Our analyses revealed that gene-content conservation and coding-sequence constraint varied differently among soybean *GPAT* subfamilies, highlighting distinct dimensions of their evolutionary divergence (**Figures 3E**, **6G**; **Supplementary Tables S5**, **S8**). Subfamily I showed strong conservation in gene content, with all assigned members belonging to core orthogroups (**Figures 3E**, **Supplementary Table S5**). Subfamily III contained the highest proportion of genes assigned to dispensable orthogroups (59.28%) and all detected proximal duplicates, suggesting a greater contribution of variable members and localized duplication to its composition (**Figures 3E**, **5B**; **Supplementary Tables S5**, **S6**). However, subfamily II exhibited the highest mean Ka/Ks ratio (0.451), whereas the ratios of subfamily III did not differ significantly from those of subfamily I (**Figure 6G**; **Supplementary Table S8**). Thus, the subfamily with the greatest representation of variable genes was not the one showing the weakest overall coding-sequence constraints (**Figures 3E**, **6G**; **Supplementary Tables S5**, **S8**). These results suggest that variation in gene occurrence across accessions can coexist with substantial conservation of the encoded proteins. Such heterogeneity is compatible with the functional diversification of plant *GPAT*s. Biochemical analyses in Arabidopsis have demonstrated differences in substrate preference and phosphatase activity among *GPAT* lineages, linking their evolutionary differentiation to the production of distinct lipid-polyester precursors (Beisson et al., 2007; Yang et al., 2010, 2012). Moreover, *GPAT4/6/8* and *GPAT5/7* perform complementary, nonredundant functions during root suberization, demonstrating that differentiated *GPAT* lineages can participate in the same physiological process through distinct biochemical contributions (Feng et al., 2022; Gully et al., 2024; Zhang et al., 2024). These findings provide a plausible functional context for the unequal evolutionary constraints observed among soybean *GPAT* subfamilies, although their specific biochemical roles remain to be established (**Figures 3E**, **5B**, **6G**; **Supplementary Tables S5**, **S6**, **S8**). Pangenome-based gene-family analysis is particularly valuable for resolving this heterogeneity because it combines orthogroup occurrence across accessions with comparative analyses of coding-sequence evolution, extending the characterization available from a single reference genome (Torkamaneh et al., 2021; Tong et al., 2025b). In the present study, this integrated perspective distinguishes variation in family membership from differences in selective constraint, providing a more informative understanding of *GPAT* subfamily evolution than either measure alone.

### Transcriptional divergence within a conserved *GPAT* co-expression network suggests potential functional partitioning

Our results suggest that transcriptional differentiation among soybean *GPAT* members occurs within a partly conserved network of expression relationships across germplasm groups (**Figures 7A, C–E**; **Supplementary Tables S9**, **S11**, **S12**). Despite the predominance of WGD or segmental duplicates and widespread purifying selection, members of subfamilies II and III were distributed across different expression clusters, indicating that evolutionary conservation can coexist with divergent tissue expression (**Figures 5B**, **6G**, **7A**; **Supplementary Tables S6**, **S8**, **S9**). This pattern is consistent with potential functional partitioning through differential regulation of related *GPAT* members. A relevant example comes from *Brassica napus*, where highly similar *BnGPAT4* homologs exhibit different expression and epigenetic profiles while retaining enzymatic activity and contributing to cuticle formation (Chen et al., 2011, 2014). The relatively high expression of several soybean *GPAT* pangenes in flowers, Leaf_bud, and pods also suggests potential involvement in lipid requirements during reproductive development. This interpretation is supported by the demonstration that loss of *ZmMs33/ZmGPAT6* disrupts anther cuticle and pollen wall development and causes male sterility in maize (Zhang et al., 2018; Zhu et al., 2019). Alongside this tissue-associated differentiation, the networks of cultivars, landraces, and wild soybeans shared 27 nodes and 25 edges, supporting the persistence of common co-expression relationships (**Figures 7C–E**; **Supplementary Table S12**). Core pangenes generally showed more stable expression across accessions, but highly connected members included both core and dispensable genes (**Figures 7B–E**; **Supplementary Tables S10**, **S12**). For example, Core7 and Dispensable23 combined high expression in flowers and pods with high connectivity in all three networks (**Figures 7A, C–E**; **Supplementary Tables S9**, **S12**). These observations suggest that variable occurrence across germplasm does not preclude conserved expression associations, supporting consideration of both categories in functional studies. Integrating pangenome-based gene-family classification with pan-transcriptome profiling therefore adds information beyond a single-reference inventory by relating gene occurrence to tissue expression, variation among accessions, and shared expression relationships. This broader perspective is consistent with previous work demonstrating the value of combined pangenome and pan-transcriptome analyses for resolving transcriptional divergence within and among gene subfamilies (Tong et al., 2025b; Liu et al., 2026; Sun et al., 2026). The potential breeding relevance is illustrated by studies showing that soybean *GmGPAT9–2* alters fatty acid composition when expressed in *Arabidopsis* seeds, while *GmGPAT1* contributes to salt tolerance in soybean (Liu et al., 2022; Li et al., 2025b). Our findings consequently identify core and dispensable GPAT candidates for future functional validation and generate hypotheses regarding their potential roles in soybean lipid traits and stress tolerance.

## Material and method

### Data sources

Genome assemblies, protein sequences, genome annotation files, and transcriptomic datasets for 29 soybean accessions were downloaded from the Soybean Multi-omics Database (SoyMD; https://yanglab.hzau.edu.cn/SoyMD) (Yang et al., 2024). Corresponding genomic resources for 1,011 plant genomes representing early-diverging plant lineages and angiosperms were obtained from the Plant Genomic Information Resources database (PlantGIR; www.plantgir.cn) (Liu et al., 2024). The Pfam profile hidden Markov model (HMM) corresponding to the acyltransferase domain (PF01553) was retrieved from the InterPro database (Blum et al., 2025).

### Identification and physicochemical characterization of the *GPAT* gene family

To comprehensively identify *GPAT* gene family members in 1,011 plant genomes and 29 soybean accessions, a combined BLASTP and hidden Markov model (HMM) based strategy was employed. Known GPAT protein sequences from *Arabidopsis thaliana* were collected and used as queries to search against the protein databases using BLASTP with an E-value cutoff of 1 × 10^-10^ (Camacho et al., 2009; Waschburger et al., 2018). In parallel, the HMM profile of the acyltransferase domain (PF01553) was downloaded from the InterPro database and used to scan all proteomes with HMMER 3.0 using an E-value threshold of 1 × 10^-10^ (Eddy, 2011; Blum et al., 2025). Candidate GPAT proteins identified by the BLASTP and HMM searches were combined, and redundant sequences were removed. All candidate proteins were subsequently submitted to InterProScan for confirmation of the conserved acyltransferase domain (Jones et al., 2014). The physicochemical properties of the confirmed GPAT proteins were calculated using the Protein Parameter Calc module implemented in TBtools II (Chen et al., 2023).

### Phylogenetic analysis of the *GPAT* gene family

Multiple sequence alignment of all identified GPAT protein sequences, together with the known GPAT proteins from *Arabidopsis thaliana*, was performed using MAFFT v7.475 (Katoh and Standley, 2013). The resulting alignment was used to construct a maximum likelihood phylogenetic tree with IQ-TREE v3.0, and the best fitting amino acid substitution model was automatically selected using ModelFinder (Kalyaanamoorthy et al., 2017; Wong et al., 2026). Branch support was evaluated using 1,000 ultrafast bootstrap replicates (Hoang et al., 2018). GPAT proteins were assigned to subfamilies according to their phylogenetic positions relative to the previously classified *Arabidopsis* GPAT proteins and the overall topology of the maximum likelihood tree.

### Pangenome classification of the *GPAT* gene family

To assess the conservation and variation of the *GPAT* gene family across the 29 soybean accessions, all identified GPAT protein sequences were clustered into orthologous gene groups using OrthoFinder v2.5.4 with default parameters (Emms and Kelly, 2019). Pangenome classification followed previously reported strategies (Tong et al., 2025b; Liu et al., 2026; Sun et al., 2026). Orthogroups present in all genomes were defined as core orthogroups. Orthogroups absent from at least one genome were classified as dispensable orthogroups. Dispensable orthogroups were further divided into three categories according to their occurrence frequency: softcore orthogroups were present in >90% but <100% of the genomes, shell orthogroups were present in 10–90% of the genomes, and specific orthogroups were present in <10% of the genomes. To characterize conserved sequence features among *GPAT* orthogroups, the longest protein sequence from each of the 47 orthogroups was selected as a representative. These representative proteins, together with the reference *GPAT* proteins from *A. thaliana*, were used to construct a phylogenetic tree as described above.

### Gene duplication type analysis

To characterize the duplication patterns of the *GPAT* gene family across the 29 soybean accessions, the whole genome protein sequences of each accession were searched against themselves using DIAMOND with an E value threshold of 1 × 10^-5^ (Buchfink et al., 2021). Genome wide duplication types were identified using the duplicate_gene_classifier program in MCScanX (Wang et al., 2012). All genes were classified as singleton, dispersed, proximal, tandem, or whole genome duplication and segmental duplicates (Wang et al., 2012). The *GPAT* genes were then extracted from the genome wide classification results and assigned to their corresponding duplication categories.

### Selection pressure analysis

To evaluate selection pressure on the *GPAT* gene family, the coding sequences and corresponding protein sequences of *GPAT* genes from the 29 soybean accessions were collected. Homologous *GPAT* gene pairs within each orthogroup were extracted from the OrthoFinder results (Emms and Kelly, 2019). The nonsynonymous substitution rate (Ka), synonymous substitution rate (Ks), and Ka/Ks ratio of each gene pair were calculated using the Simple Ka/Ks Calculator module in TBtools II (Zhang et al., 2006; Chen et al., 2023). Gene pairs without valid Ka or Ks estimates were excluded from subsequent analyses.

### Transcriptome data processing and expression analysis

Transcriptomic expression data for the 29 soybean accessions were obtained from the Soybean Multi-omics Database (SoyMD; https://yanglab.hzau.edu.cn/SoyMD) (Li et al., 2025a). *GPAT* genes were assigned to their corresponding orthologous groups according to the OrthoFinder results, and the expression data were subsequently organized at the pangene level for the 47 *GPAT* orthogroups (Emms and Kelly, 2019). For each *GPAT* pangene and tissue, mean TPM values and standard deviations (SDs) were calculated across soybean accessions in which the corresponding pangene was present. Accessions lacking the pangene at the genomic level were treated as missing values and excluded from the calculation of mean TPM, SD, and relative SD, whereas TPM values of zero were retained when the pangene was present but showed no detectable expression in a given tissue. Mean expression values were transformed using log2(mean TPM + 1) before downstream analysis (Tong et al., 2025b). Hierarchical clustering of the 47 *GPAT* pangenes was performed using Euclidean distance and complete linkage, and the resulting expression clustering tree was compared with the *GPAT* phylogenetic tree to evaluate transcriptional divergence within and among subfamilies. The expression matrix used for the final heatmap therefore represents log2(mean TPM + 1) values across the eight tissues.

### PCC-based co-expression network analysis

To investigate the co-expression relationships among soybean *GPAT* pangenes, expression data from 29 soybean accessions across eight tissues were organized at the pangene level according to the 47 *GPAT* orthogroups. Pearson correlation coefficient (PCC) analysis was performed separately for cultivars, landraces, and wild soybeans using custom Python scripts. Before correlation analysis, expression values were transformed using log2(x + 1). Pairwise PCC values were calculated between *GPAT* pangenes within each germplasm group, and strongly correlated pairs with |PCC| ≥ 0.95 were retained to construct separate co-expression networks (Zainal-Abidin et al., 2022). Nodes represented *GPAT* pangenes, while edges indicated positive or negative expression correlations. Network visualization and topological analyses were performed using Gephi (Mathieu Bastian and Jacomy, 2009).

### Data processing

All statistical analyses and figures were performed using custom Python scripts.

## Data Availability

The original contributions presented in the study are included in the article/**Supplementary Material**. Further inquiries can be directed to the corresponding author.
